# Quantifying knowledge from the perspective of information structurization

**DOI:** 10.1371/journal.pone.0279314

**Published:** 2023-01-04

**Authors:** Xinbing Wang, Huquan Kang, Luoyi Fu, Ling Yao, Jiaxin Ding, Jianghao Wang, Xiaoying Gan, Chenghu Zhou, John E. Hopcroft

**Affiliations:** 1 Shanghai Jiao Tong University, Shanghai, China; 2 State Key Laboratory of Resources and Environmental Information System, Institute of Geographic Sciences and Natural Resources Research, Chinese Academy of Sciences, Beijing, China; 3 Cornell University, Ithaca, New York, United States of America; University of Siena, Italy, ITALY

## Abstract

Scientific literature, as the major medium that carries knowledge between scientists, exhibits explosive growth in the last century. Despite the frequent use of many tangible measures, to quantify the influence of literature from different perspectives, it remains unclear how knowledge is embodied and measured among tremendous scientific productivity, as knowledge underlying scientific literature is abstract and difficult to concretize. In this regard, there has laid a vacancy in the theoretical embodiment of knowledge for their evaluation and excavation. Here, for the first time, we quantify the knowledge from the perspective of information structurization and define a new measure of knowledge quantification index (KQI) that leverages the extent of disorder difference caused by hierarchical structure in the citation network to represent knowledge production in the literature. Built upon 214 million articles, published from 1800 to 2021, KQI is demonstrated for mining influential classics and laureates that are omitted by traditional metrics, thanks to in-depth utilization of structure. Due to the additivity of entropy and the interconnectivity of the network, KQI assembles numerous scientific impact metrics into one and gains interpretability and resistance to manipulation. In addition, KQI explores a new perspective regarding knowledge measurement through entropy and structure, utilizing structure rather than semantics to avoid ambiguity and attain applicability.

## Introduction

With the growth of academic big data, the contradiction between the ability of human knowledge acquisition and the speed of information generation is increasingly prominent [1]. Nowadays, academic literature has entered an explosive growth period with further increased scientific research investment [2,3]. While the large volume of scientific papers might produce some ground-breaking knowledge, it also places researchers in the dilemma of reading fatigue [4–6]. This predicament may be still sustainable for the newly emerging disciplines, but for disciplines that have undergone long-term development, the requirements for researchers to conduct research are demanding. Given the increasing interest in alleviating the burden of literature research for scientists, we ask: Can we untangle the role of knowledge from productivity and ease reading fatigue? This question drives us to develop a new metric to quantify the knowledge amount of scientific productivity in multiple disciplines.

Productivity, representing the number of publications by an individual or group within a specified period, is a frequently used metric to gauge a scientist’s or scientific community’s performance. Many quantitative indicators have been proposed around productivity either from article-level, author-level, venue-level, or institution-level, to characterize their respective scientific impact (see Table 1 for a summary). As can be seen from Table 1, citations [7–10], the epitome of scientific influence, and those citation-based measures like h-index [11], g-index [12], and impact factors [13], can help researchers screen out influential literature from different perspectives of papers, authors and journals. While those measures advantage in the intuitive judgment of productivity, they are simply statistical indicators based on the citation quantity that focuses on the portrayal of direct attention [14], falling short of reflecting how knowledge inspires new knowledge between different published scientific articles. h-index tells that at least h papers have each been cited h times, g-index tells that the top g papers receive together at least g^2^ citations, and impact factor tells the yearly mean number of citations of papers published in the last two years in a given journal. Besides, Relative Citation Ratio [15], Field-weighted Citation Impact, and Source Normalized Impact per Paper weight the citations relying on the fields that the paper belongs to. We note that these metrics, established on basic mathematical combinations which are only affected by direct citations, cannot reflect the importance of a paper’s location in the citation network, that is, they are far from the knowledge that should be position-sensitive in a structure and perturbed by any change in the citation network.

**Table 1:** Metrics of science impact.

	Category	Description
**Article-level**		Characterized by KQI of nodes in a citation network.
Citation-based	Citation	The number of times an article is cited by other articles, books, etc.
	Relative Citation Ratio [15]	The field-normalized citation compared with the other papers that appear alongside it in reference lists.
	Field-weighted Citation Impact	The field-normalized citation compared with the total citations that would be expected based on the average of the subject field.
Network-based	PageRank	The likelihood that a reader randomly following references will arrive at any particular article.
**Author-level**		Characterized by aggregated KQI of nodes with the same author in a citation network.
Citation-based	h-index	The maximum value of h such that the given author has published at least h papers that have each been cited at least h times.
	i10-index	The number of publications with at least 10 citations.
	g-index	The maximum value of g such that the top g articles received together at least g^2^ citations.
	Author Impact Factor	The mean number of citations given by papers published in year y to papers published by author in a period of Δy years before year y.
Network-based	PageRank-Index [16]	The individual percentile ranking of a scientist based on cumulative weighted contribution using PageRank.
	Author-level Eigenfactor	The eigenvector centrality in the network by regarding authors as nodes in a network of citations.
	Erdős number	The collaborative distance between mathematician Paul Erdős and another person.
**Journal-level**		Characterized by aggregated KQI of nodes with the same journal in a citation network.
Citation-based	Impact Factor	The yearly mean number of citations of articles published in the last two years in a given journal.
	CiteScore	The yearly mean number of citations of articles published in the last four years in a given journal.
	h5-index	The h-index for articles published in the last 5 complete years.
	Source Normalized Impact per Paper	The contextual citation impact by weighting citations based on the total number of citations in a subject field.
Network-based	Eigenfactor	The eigenvector centrality in the network similar to PageRank, weighted by journals’ importance.
	SCImago Journal Rank	The variant PageRank considering connections to high-scoring nodes to contribute more.
**Affiliation-level**		Characterized by aggregated KQI of nodes with the same affiliation in a citation network.
Human-based	QS	The annual publication of university rankings by Quacquarelli Symonds considering teaching, research, nurturing employability, and internationalization.
	THE	The annual publication of university rankings by Times Higher Education magazine considering teaching, research, citations, international mix, and industry income.
	ARWU	The annual publication of university rankings by Shanghai Jiao Tong University considering teaching, research, faculty, and resources.
Count-based	Nature Index Top institutions	The scientific output of institutions.
**Country-level**		Characterized by aggregated KQI of nodes with the same country in a citation network.
Count-based	SCImago Country Rank	The scientific output and citations of countries.
	Nature Index Top countries	The scientific output of countries.

Considering that the citation-based approach lacks the ability to capture localized characters in structure, we hope to utilize citation relations and network-based methods to estimate knowledge. Although the importance of all the papers and their references can be uneven, the citation network may help us understand the knowledge embedded in the overall scientific discourse. The state-of-the-art network-based measures like PageRank [16–20], eigenfactors [21], and SCImago Journal Rank, leveraging iterations to achieve stability, use the probability of arriving at a particular article to represent the scientific impact. These measures take into account the importance of the topological locations and succeed in sourcing high-impact papers, authors, or journals from citation networks. However, these measures do not tell us clearly how and to what extent the probability distribution from a random walk reflects knowledge. Thus, we still lack a quantitative metric to reflect the knowledge contained in scientific productivity, which can further help understand the knowledge value of articles, reveal the development of academic knowledge brought about by the expansion of the discipline, and further alleviate this contradiction between knowledge acquisition and information generation.

Although the measurement of knowledge is important, the definition of knowledge, especially the quantification of knowledge, remains largely unexplored. Plato once proposed the JTB theory (the view that knowledge is justified true belief) thousands of years ago [22], although Gettier questioned it [23], and since then there is no accurate definition of knowledge. Numerous studies have shown the structure of knowledge [24,25], and the important role of the network in explaining knowledge [26]. Considering a large number of association relationships in academic data, such as citations, we modeled these in an academic citation network [7] and tried to find knowledge in this structured space. Coincidentally, some indications of knowledge are seen in the citation network: **(1)** The subsequent citations of the paper reflect the widespread recognition of the paper, i.e. relatively **truth** [27] in the network, also similar to the "the relativity of knowledge" [28,29] proposed in the epistemology. **(2)** The literature references also reflect whether the source of the paper is reliable and **justified**. Therefore, knowledge can be expressed as the paper and the structure on which it depends, though it may be hard to understand. In other words, the effect brought by knowledge is embodied in the association of academic networks. We cannot tell knowledge that is not belonging to the structure, just like we cannot tell a node that is outside of a topological network. Based on this assumption, we present several characteristics of knowledge in the evolutionary network:

- Knowledge has strict hierarchies and no circular reasoning: Although this puts forward higher requirements on the reliability of data sources, the data in the knowledge domain is often of higher quality, which is different from the general big data.
- Knowledge evolves over time: Knowledge is not fixed, which means that new knowledge is constantly being included, to refresh the existing knowledge structure.
- Some knowledge may fade over time [11,30]: The aging of knowledge is manifested as the decline of the inheritance relation between new knowledge and old knowledge. That is, it cannot be recalled from present knowledge of its origin.
- Structure matters more than the literal meaning of knowledge: The value of knowledge depends more on its position in the knowledge structure than on its content. And the structure is more objective than the content.

In 1989, Ackoff introduced the framework of the DIKW pyramid [31] (data, information, knowledge, and wisdom) and described the positioning of knowledge. The DIKW pyramid lays a very instructive foundation for our knowledge measurement. DIKW implies that knowledge stems from information, and the quantification of information has quite mature theories, such as Shannon’s entropy [32], Angsheng Li’s structural entropy [33], etc. Therefore, information theory can serve as a clue to the quantification of knowledge. In physics, entropy measures the extent of disorder [34], and knowledge organizes disordered data into ordered data [35,36]. Shannon entropy [32] measures the extent of disorder in the discrete probability distribution, while structural entropy [33] measures the extent of disorder after organizing the discrete one into a structured network. The above two exactly correspond to the process of knowledge turning disorder into order. Therefore, the difference between the two entropies is the role of knowledge in it.

Here, we present Knowledge Quantification Index (KQI) metric, a quantitative knowledge index based on a citation network, to reflect the extent of disorder difference (knowledge amount) caused by structure (order). We collected 214 million articles published from 1800 to 2021, and establish a citation network to calculate KQI for articles, authors, affiliations, and countries (S1-S3 Tables and S1 Fig in S1 File). We demonstrate KQI’s effectiveness in identifying valuable knowledge compared with traditional metrics. Our finding introduces a new way of measuring knowledge from a structural perspective, thus circumventing the ambiguity caused by semantics and thus achieving wider applicability. Furthermore, despite the dizzying array of scientific impact metrics (Table 1), KQI takes all those in one.

## Results

### Quantifying knowledge from information

Knowledge can be described as what is annihilated during the process of structuring information. The structuring here is a process of transitioning from discrete information to interrelated information. Since we cannot directly quantify this process, a natural idea is to quantify the information separately at the beginning and end of the structuring, just like using the buoyancy method to measure the bulk of an irregular object. This structuring is discussed under the category of directed acyclic graphs, because of the knowledge structure of ideal citation networks. A citation network before structuring corresponds to discrete nodes, and after structuring corresponds to the community of hierarchies composed of them. For the beginning state, Shannon Entropy [32] *H*^1^ has profound foundations for the measure of discrete categorical information, which takes degree distribution as probability distribution and is denoted as

$$H^{1}=\sum -\frac{d^{in}}{m}\log\frac{d^{out}}{m},$$ (1)

where *m* is the number of edges, *d*^*in*^ and *d*^*out*^ are the in-degree and out-degree of a node. For the ending state, structural entropy [33] is capable of quantifying the entropy of networks, relying mainly on the partitioning tree *T*, which is a kind of hierarchical partitioning community like a postal code. Each node *α* on the partitioning tree represents a community, which is a set of nodes, and in this way divides the edges of the network into four groups: inner edges (or volume) *V*_*α*_, outgoing edges *g*_*α*_, incoming edges, and outer edges. Such that, the structural entropy *H*^*T*^ is defined as

$$H^{T}=\sum_{\alpha \in T,\alpha \neq \lambda }-\frac{g_{\alpha }}{2m}\log\frac{V_{\alpha }}{V_{\alpha^{-}}},$$ (2)

where *α*^−^ represents the parent node of *α* in the partitioning tree and *λ* is the root node.

**Fig 1:**
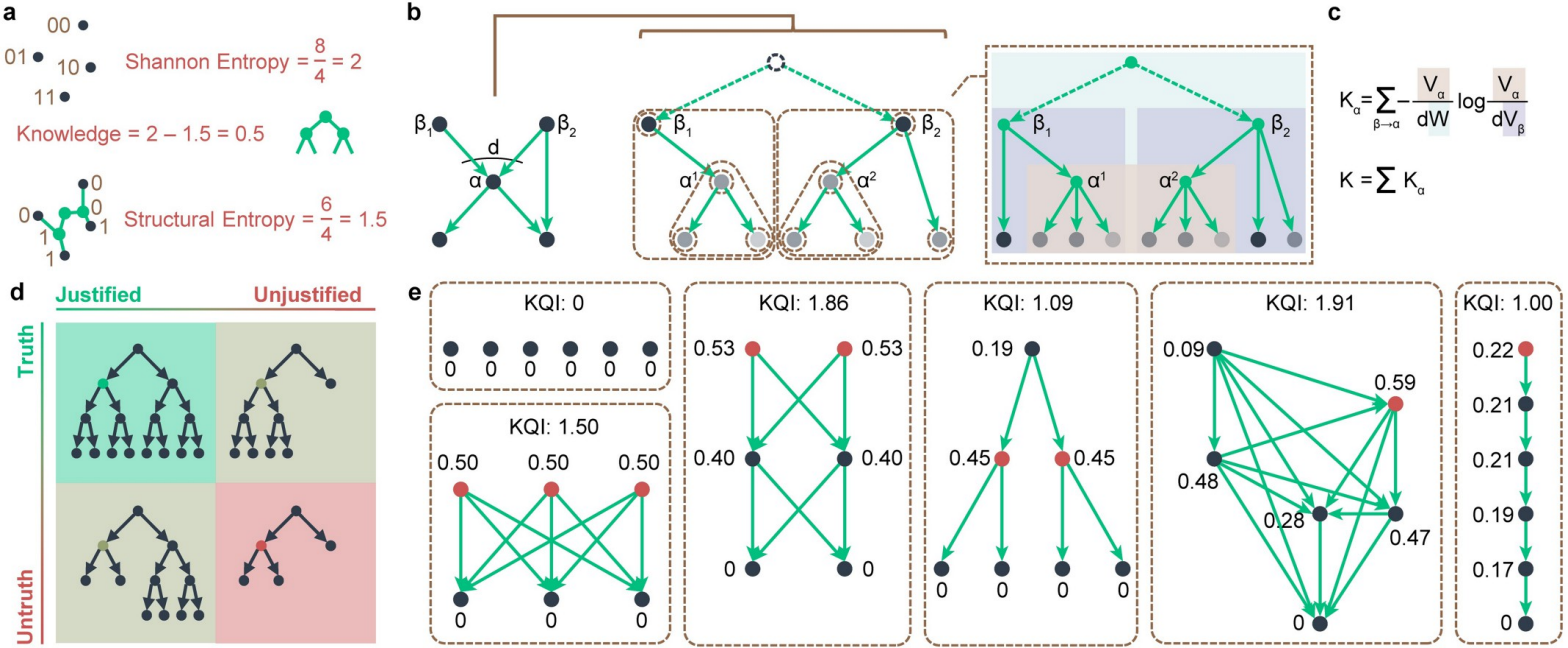
Quantifying knowledge with KQI. **a,** Three information-related quantitative indicators. Shannon entropy encodes a discrete probability distribution, where it takes an average of 2 bits to uniquely identify an object. Structural entropy takes structure into account as opposed to Shannon entropy, and it takes an average of 1.5 bits because of the shared encoding caused by the structure. The difference between these two entropies is precisely the difference caused by structure, namely the KQI. **b,** Knowledge tree, decomposing process, and transformation to partitioning tree. Multiple trees can be split from a knowledge tree, with volume assigned (color depth of the node). The nodes that break up eventually become fragments with less weight. Trees imply the structure of layered communities (brown dotted circle), i.e., the partitioning tree (tree in the brown dotted box). The green nodes in the partitioning tree imply the community rather than the actual nodes. **c,** KQI formula. α in the formula corresponds to the node in **b**, although it later splits into two fragments, and β corresponds to the parent nodes of α. V represents the volume (number of edges) of the subtree, corresponding to the shadows in **b**. W is the volume of the entire graph. **d,** KQI-JTB matrix. For any knowledge, volume means truth, and the difference from parents tells whether it is justified. Knowledge of maximum KQI (green) should be justified truth. Knowledge with at least one of truth and justification comes next, i.e., unjustified truth or justified untruth. Unjustified untruth has little knowledge (red). **e,** KQI of typical topologies. Nodes with the highest KQI are marked in red.

Now that we have defined the beginning status (Shannon entropy *H*^1^) and the ending status (structural entropy *H*^*T*^) of the structuring, KQI is built on information theory and calculated by the subtraction between these two entropies, to quantify the knowledge *K* lurking in citation networks (Fig 1A):

$$K=H^{1}-H^{T}.$$ (3)

Here, the knowledge *K* is always greater than 0, which is further concluded from Eq (4) and S3 Text in S1 File. This is consistent with the fact that the structuring process reduces entropy.

Considering the knowledge structure implied in the citation network, here, the partitioning tree is replaced by the actual structure of knowledge: any knowledge is either inferred from existing knowledge (belongs to the parent knowledge community) or is a pure axiom. However, unlike the partitioning tree with only one root community [33], the structure of knowledge can be seen as a combination of many partitioning trees, because we have much axiomatic knowledge, and these partitioning trees overlap each other because a piece of knowledge can be inspired by multiple knowledge, i.e. belong to multiple knowledge communities simultaneously. We modeled this kind of structure as a knowledge tree (Fig 1B). Under the assumption of such a knowledge structure, we formulate KQI explicitly (Fig 1C, S2 Text in S1 File). Under the assumption of such knowledge structure, denoting *β* as a parent of *α*, *d* as the number of parents of *α*, *W* as the graph size, *V*_*α*_ as the volume including all descendants of *α*, we extended Eq (3) to express KQI explicitly as

$$K=\sum K_{\alpha },\;K_{\alpha }=\sum_{\beta \to \alpha }-\frac{V_{\alpha }}{dW}\log\frac{V_{\alpha }}{dV_{\beta }}.$$ (4)

The further rigorous derivation is listed in the S3 Text in S1 File.

Based on this formula, KQI is related to acceptability and dependability, both of which originate from truth and justification in JTB theory [22] (Fig 1D). Acceptability refers to whether knowledge is recognized, i.e., how much knowledge is inherited directly or indirectly from that knowledge. Dependability refers to whether the source of knowledge is equally or more recognized, i.e., how fully the parents can support the generation of the knowledge. Acceptability and dependability, elements of scientific knowledge [37,38], correspond to the first and second terms of Eq (4) (see *Methods*, Fig 1C). Therefore, KQI exactly captures the crucial nodes in a network, which is intuitive for us (Fig 1E).

### Knowledge evaluation on academic data

Utilizing Acemap [39] academic database, we retrieved and integrated known academic sources, including but not limited to Nature, Science, Elsevier, and Springer, and collected 214 million journal articles and conference papers from 1800 to 2021, covering 292 fields in 19 disciplines. More details are provided in **Methods.** We created citation networks for these academic data and used the proposed KQI metric to measure the knowledge in the network.

**Fig 2:**
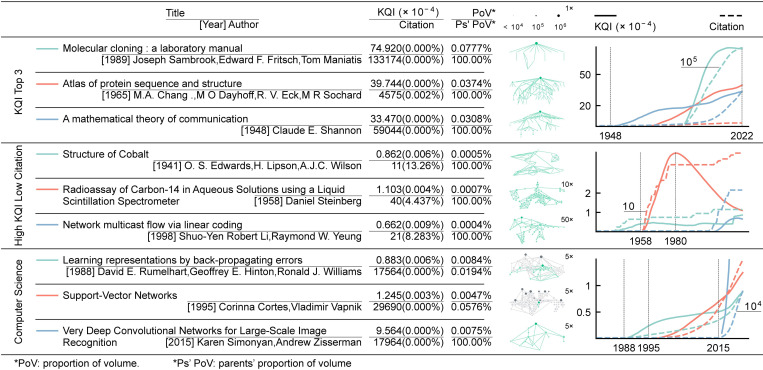
KQI, citation, volume, and visualization of typical literature. Three categories, overall KQI top 3, high KQI low citation, and computer science field, each containing three papers, are listed with KQI (ranking percentage), citation (ranking percentage), the proportion of volume, and parents’ proportion of volume. The position of each paper P in the citation network is also visualized, where the dark green node is the paper P itself, the light green nodes are inherited from P, the dark gray nodes are referenced by P, and the light gray nodes are inherited from these references. The size of the nodes indicates the volume, and the pictures where the volume of child nodes is small are enlarged by 5x, 10x, and 50x to show more details. The trends in KQI (solid) and citations (dashed) are also plotted over time, from the publication of the paper to the present.

KQI can be used for reflecting the knowledge attribute of the paper, i.e., the acceptability and dependability as mentioned above. The higher the value of KQI, the stronger the two knowledge attributes are, i.e., high KQI papers are considered as justified truth, deriving from reliable parent knowledge and spawning numerous child knowledge (Fig 1D). Therefore, we use KQI to rank papers, which reflects the above characteristics of the papers (S1 Table in S1 File). The top KQI-ranked papers are classics with high reputations in the scientific community, such as *Molecular cloning*: *a laboratory manual* (MCLM), *Atlas of protein sequence and structure* (APSS), *A mathematical theory of communication* (MTC), etc. (Fig 2). Among them, MCLM is almost an indispensable laboratory manual and reference in the field of molecular biology, with no other manual being as popular as it has been for decades; and APSS is even a more classic monograph on bioinformatics. MTC is the masterpiece of Claude Shannon, the father of information theory, and influenced later communication, linguistics, and cryptography profoundly. These three papers spawned numerous papers in terms of acceptability, and none of them cited other references, i.e., they were groundbreaking axiomatic knowledge, in terms of dependability. Specifically, defining the acceptability of a paper P as the ratio of volume to overall, and the dependability as the ratio of acceptability between P’s references and P, the results indicate that papers with high KQI rankings have significantly higher acceptability and dependability than those with high citation rankings (Fig 2).

**Fig 3:**
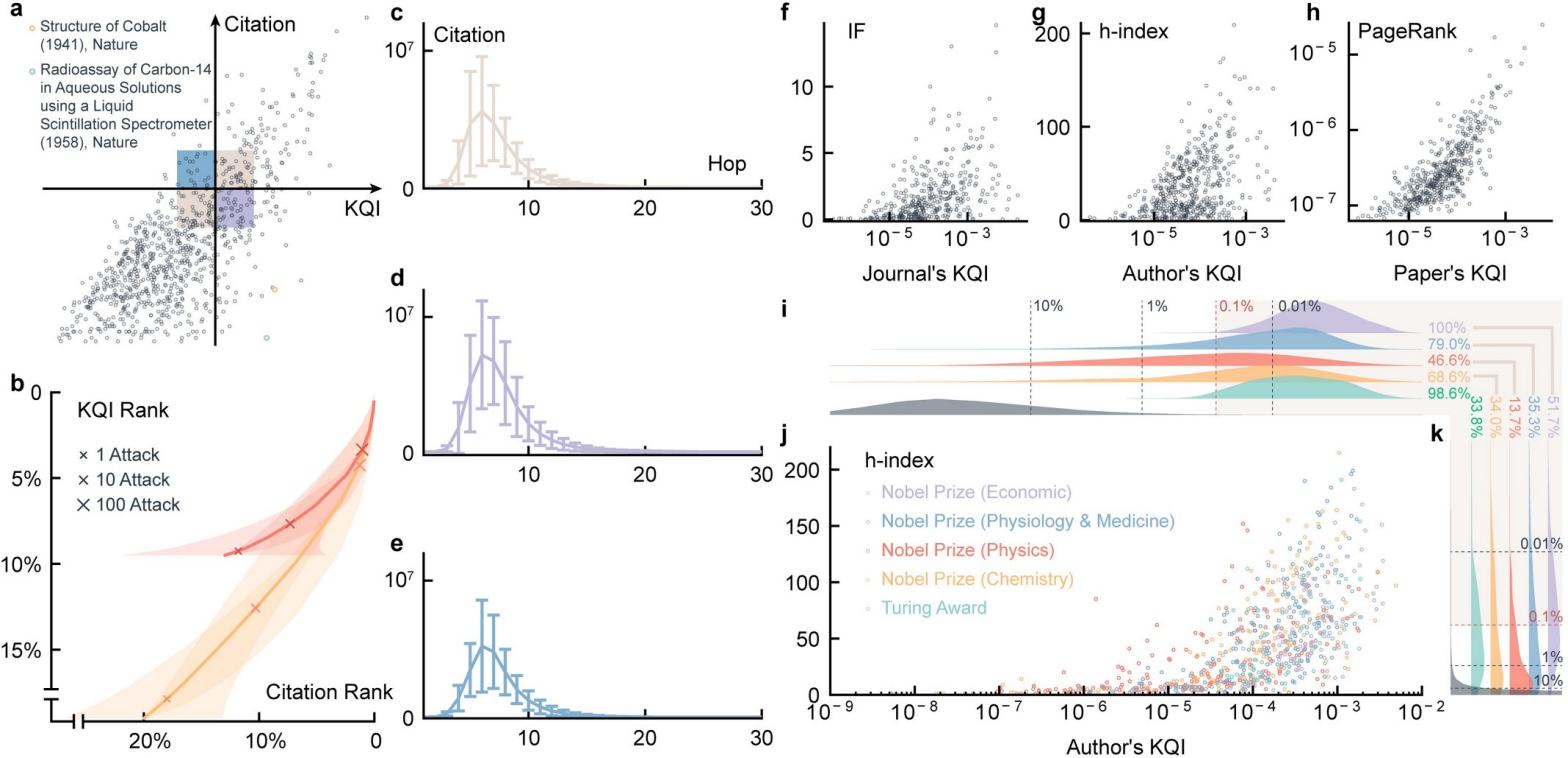
KQI-based ranking and comparisons. **a,** Quadrants of KQI and citation. The scatter diagram shows the triangle at the lower right, which implies that higher citations correspond to higher KQI, but lower citations do not mean lower KQI. Two papers shown in the delta quadrant give examples where valuable literature receives few citations. **b,** The impact of artificial attacks on rankings. Plot the change curve of KQI and citation rankings by deliberately citing certain articles, adding 1,10,100, or more citations, respectively. The shadow represents the standard deviation of the sample. After the attack, the citation ranking significantly changes more than the KQI ranking. **c-e,** Citation changes for different hops. By sampling the three shaded areas in **a**, the number of papers derived from these papers with different hops is shown. It can be observed that articles in region **d** have a greater aftereffect, while those in region **e** have a lower aftereffect. **f-h,** Comparison among h-index, impact factor, PageRank, and KQI [40,41]. The scholars with high citations are more inclined to have higher KQI, while the impact factor of the journal has little relationship with its KQI. KQI and PageRank show a significant positive correlation. **i-k,** Laureates distribution. The h-index and KQI statistics of Nobel Prize winners (Economics, Physiology & Medicine, Physics, Chemistry) and Turing Award winners show that KQI can better distinguish laureates from ordinary authors (grey). 100% of Nobel Prize winners (Economics) are ranked in the top one-thousandth of KQI, while 51.7% are ranked in the top one-thousandth of h-index. Other results are similar.

KQI helps excavate valuable papers even if they are not highly cited, which assists in easing reading fatigue among researchers. Statistics of our collected journal articles and conference papers from 1800 to 2021 show that the number of literature today is three times that of 20 years ago and fifteen times that of 50 years ago (S5a Fig in S1 File). By classifying literature into 292 sub-disciplines of concern to researchers, 39% of sub-disciplines have over 1 million articles, and 99% have over 100 thousand articles, with which researchers are overwhelmed (S5b Fig in S1 File). Papers with different knowledge and citations can be divided into four quadrants: high knowledge and high citations, low knowledge and low citations, high knowledge and low citations, low knowledge, and high citations. Citations are qualified to the first two states, and KQI encompasses all of them. For example, *Structure of Cobalt*, by Arthur Wilson, published in Nature in 1941, advanced the study of X-rays and is still regarded as the authority for his great contributions to the X-ray field, but only 11 citations were received (Figs 2 and 3A). The assay proposed by Daniel Steinberg in *Radioassay of Carbon-14 in Aqueous Solutions using a Liquid Scintillation Spectrometer* (RCASLSS), published in Nature in 1958, is still the preferred method for detection of carbon-14, but only 40 citations were received (Figs 2 and 3A). In addition, the top KQI ranked paper in the discipline of network coding: *Network multicast flow via linear coding* (NMFLC), from Raymond W. Yeung who made a pioneering contribution to the field of network coding, was published in 1998 at the International Symposium on Operations Research and its Applications in engineering, technology, and management (Fig 2). Despite the low citations, NMFLC has produced some high-impact papers, such as *Linear network coding*, *An algebraic approach to network coding*, and *Network coding theory*, which received about 4,000, 3,000, and 300 citations respectively, and underpinned the development of modern communication technologies. Because high-impact papers all cite this paper, it appears to be of considerable value. In fact, most papers with high citations usually receive fair assessment, while the papers with low citations are often undervalued, which can be detected by KQI (Fig 3A).

Besides, KQI varies with the shifting of research hotspots. Taking the computer science discipline as an example, Connectionists represented by backpropagation algorithms originated in the 1980s but were eclipsed by the emergence of Analogizers represented by support vector machines around 1995, and flourished with deep learning in recent years (Fig 2). In addition, the information theory represented by MTC continues to be favored by the scientific community to date, while the KQI of RCASLSS reached its peak in 1980 (Fig 2).

To make the results more convincing, we aggregate the KQI of the papers by author, and then give a ranking of the authors. Thanks to the additivity of entropy, our aggregation is just to add up the KQI of all the papers of an author, and interestingly, the authors at the top of the list are influential. To eliminate subjective factors, the Turing Award and Nobel Prize, which are highly recognized in the academic circle, are selected as the evaluation criteria. We find that these laureates have significantly higher KQI than other authors, while the h-index is far from being able to achieve this effect (Fig 3G and 3I–3K, S3 and S4 Figs in S1 File). Many laureates do not have a high h-index, such as Edwin Catmull, Raj Reddy, Ken Thompson, and so on. Besides the two well-known awards, many famous authors are returned with top KQI ranking, such as the father of information retrieval Gerard Salton, the father of information theory Claude Shannon, and so on (S2 Table in S1 File). Then based on the ranking of papers, we also rank the affiliations and countries. Among affiliations with high KQI, the top 20 are not with China, and the number of literature and KQI of the United States both far exceed those of other countries (S3 Table in S1 File). Nowadays, China has almost half as much literature as the United States, but still lags far behind in the KQI (S1 Fig). This is also in response to a shift in China’s scientific research in recent years from quantity to quality.

**Fig 4:**
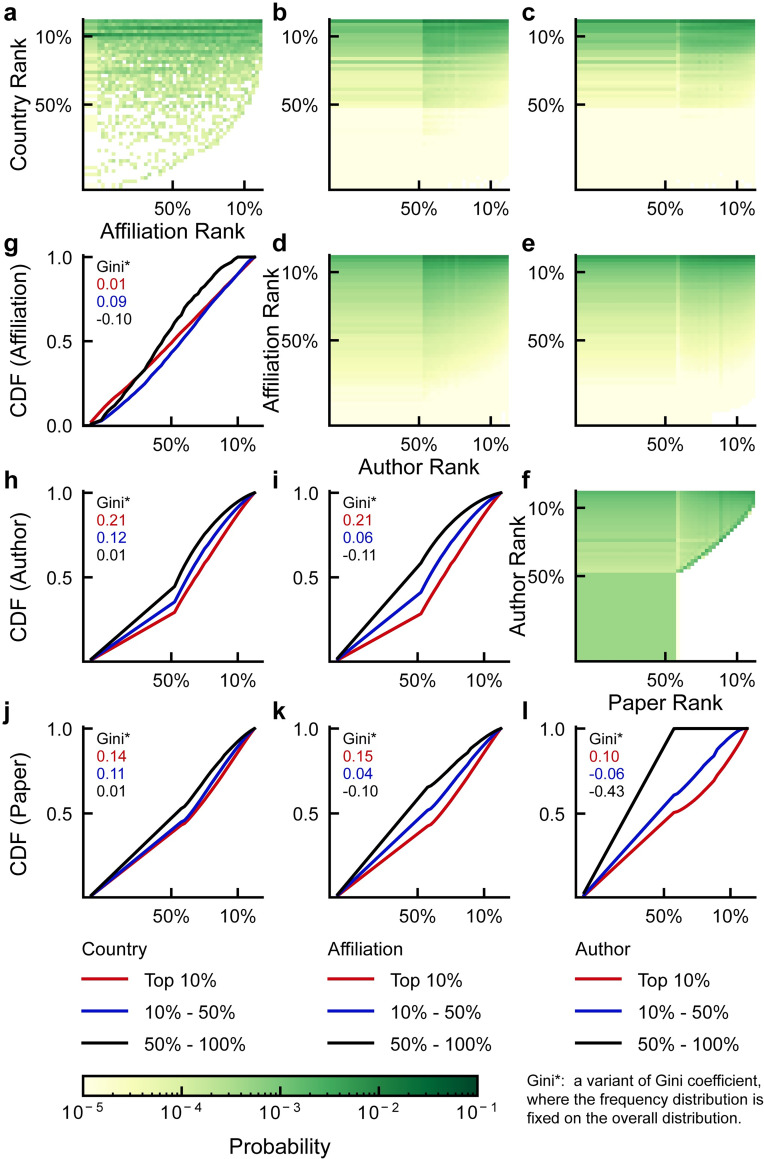
Cross comparison among countries, affiliations, authors, and papers based on KQI ranking. **a-f,** the 2D probability distribution of pairwise combinations. The axes represent the position of the KQI ranking in specific categories, where a smaller number indicates a better rank. The darker the color, the higher the probability. **g,** Cumulative distribution of affiliations across different ranking ranges of countries. **h-i,** Cumulative distribution of authors across different ranking ranges of countries and affiliations. **j-l,** Cumulative distribution of papers across different ranking ranges of countries, affiliations, and authors. A variant of the Gini coefficient indicates the inequality of quality, from -1 to 1, representing the distribution of the worst quality to the best quality (see *Methods*).

As KQI is widely applicable to countries, affiliations, authors, and papers, we make a cross-comparison among them and use a variant of the Gini coefficient (see *Methods*) to characterize the inequality of the distribution for exploring their potential dependencies. We find that countries with high KQI rankings such as the US, UK, Germany, Japan, and China, typically have much more affiliations (Fig 4A) and their distributions of affiliations on KQI rankings are more balanced (Fig 4G), i.e., they have a more well-rounded deployment of affiliations, not focusing only on quantity regardless of quality, or only on the productive affiliations. Consistent with intuition, the level of both countries and affiliations shows a positive correlation with the number of authors and papers (Fig 4B–4E), and countries and affiliations with high KQI rankings also have relatively higher quality groups of authors and papers (Fig 4H–4K). In addition, the level of scientists is little related to the number of papers they publish (Fig 4F) but strongly correlated with the quality of their publications, where high-ranked scientists have notably larger Gini*, which implies a higher proportion of high-ranked papers (Fig 4L). Intuitively, countries that concentrate all their efforts on developing productive affiliations may lead to higher rankings, but the high-ranked countries deploy their affiliations reasonably well balanced. Affiliations can easily recruit more scholars to raise their productivity, but we also note a preference for high-ranked authors and papers amongst the high-ranked affiliations. This difference is even more pronounced for authors, for whom improving the quality rather than quantity of their papers is essential to improving their KQI rankings. This also implies the potential of KQI to eliminate publication bias and break the loop of the “rat race”.

## Discussion

Among many complicated scientific impact metrics (Table 1), the most representative and commonly used four: citation count, h-index, impact factor, and PageRank, were selected for detailed comparison with KQI. Most of the other metrics can be regarded as the derivation of these four, with a similar nature. For example, Relative Citation Ratio and Field-Weighted Citation Impact are mathematically weighted citation counts, i10-index and g-index make minor changes to the selection of papers for h-index, CiteScore is an impact factor over different time ranges, and Eigenfactor is a weighted PageRank of some kind.

**Fig 5:**
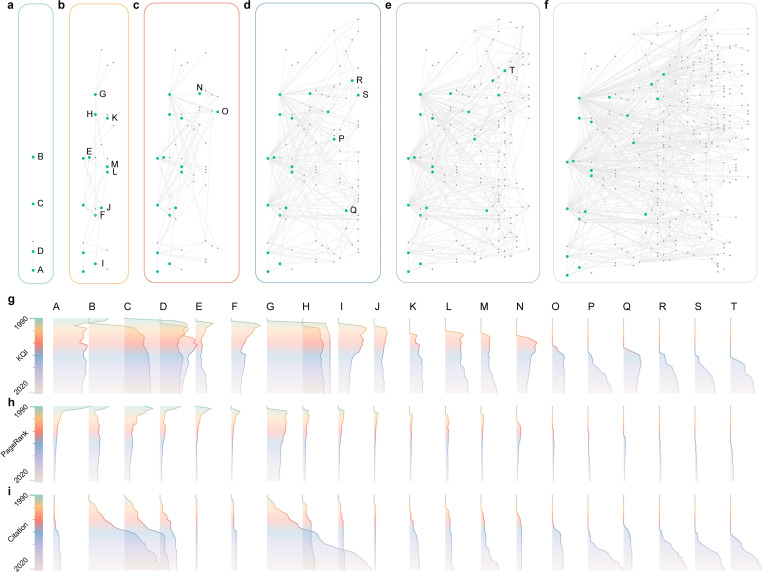
KQI changes with the evolutional network. **a-f,** Simplified evolutional network on informetrics field from 1990 to 2021. Nodes represent the articles and edges represent the citation relationships. The nodes at the right cite the nodes at the left, in chronological order from left to right. The networks correspond to snapshots in 1990, 1995, 2000, 2005, 2010, and 2020 respectively. Additional 20 nodes (green) are marked to specifically compare with changes in metrics. **g-i,** KQI, PageRank, and citation variation of typical nodes. The gradient color corresponds to the evolution time of **a-f**. F, I, N, Q represent articles that gradually drifted away from the research center, with reduced KQI, reduced PageRank, and smooth citations. E, J represent articles that are little-known but enlightening, with considerable KQI and few citations. A, C, D shows that PageRank of articles whose citations do not continue to grow decreases, while the KQI stays high. B, G shows that increasing citations maintain PageRank. D, E, M shows that KQI reflects the change of research hotspots in the network.

KQI is essentially built up based on citation relationships but is different from citations. Specifically, there are four differences between them:

1. **Citations only focus on the number of references to one paper, while KQI considers the citations of all derived papers.** This is the benefit brought by the introduction of volume. Even if the citation of a paper is not high, if it produces some influential papers, it will indirectly explain the value of this paper (Fig 3A and 3C–3E).
2. **Citations will only increase, and the influence of the paper will only grow. But, KQI will increase or decrease dynamically with time, which reflects the knowledge content of the paper at a specific time.** Although the graph structure changes with more and more nodes and edges, the KQI of different parts receives a quite different promotion or attenuation as leaning toward or deviating from the crucial points. Therefore, the KQI of a certain paper may increase or decrease, affected by the latest research hotspots (Figs 2 and 5A–5G).
3. **Citations only reflect the truth of a paper. KQI considers the truth and belief of a paper.** Like JTB theory, truth is reflected by citations because it is widely accepted, and belief is reflected by papers’ references, which tells if the paper is based on some widely accepted paper. The KQI’s formula considers both two (Fig 1C and 1D).
4. **Citations are easy to be manipulated, and manipulating KQI is even more difficult.** Due to the inevitable fake citations, in reality, it is easy for powerful people to solicit many cheap citations to show their influence. But they do not much damage to the KQI, because the KQI depends on the structure of the entire citation network unless you have the power to break it. KQI is better at resisting attacks than citations (Fig 3B).

Researchers have proposed that the role of the h-index is equivalent to citations [41] and still bound by citations, although the h-index measures both the productivity and citation impact of a scientist. We find a weak correlation between the h-index and KQI. For high h-index scholars, KQI is usually not too bad (Fig 3G and 3I–3K). However, compared with their h-index, KQI corrects those scholars who exploit the h-index loophole to a certain extent. Besides, the h-index often buries some outstanding scholars, such as the mentioned Turing Award and Nobel Prize winners, which are included in KQI.

Also, researchers questioned the impact factor for abuse [40], although the impact factor is frequently used as a proxy for the relative importance of a journal within its field. Our experiment confirms that the impact factors of journals have a limited role in determining the value of their published papers, which fits our intuition. It can only be inferred those journals with larger impact factors are less likely to receive bad articles, but it cannot be inferred those journals with smaller impact factors have no valuable articles (Fig 3F). This has considerable guiding significance for us. There is no need to be obsessed with authoritative journals. The quality of the articles should not be evaluated directly by the level of the journal, but by the value of the article itself.

PageRank [16,20] takes into account the structure of the network in comparison with the h-index and impact factor, but it is still not exactly the same as KQI (Figs 3H, 4G and 4H). As a traditional method to measure the importance of nodes in a graph, PageRank performs a random walk on the graph to rank nodes by their information flow. However, this method only tells the popularity, which is unequal to the knowledge. We are more interested in the quantity, value, and minimum redundancy of knowledge. To be more specific, KQI is better than PageRank in the following aspects:

1. **Interpretability.** PageRank is just a state of balanced information flow, which expresses influence and lacks interpretation at the knowledge level. KQI expresses the structure reflected by the difference between Shannon entropy and structural entropy, which is related to the meaning of knowledge (Fig 1A).
2. **Formulation.** PageRank can be viewed as a subset of KQI, that is, PageRank expresses a similar meaning to the volume variable *V* mentioned in Eq (4) (see *Methods*, Fig 1B and 1C). In this sense, KQI is more advanced than PageRank.
3. **Complexity.** The algorithm complexity of PageRank depends on the number of iterations required to achieve convergence, while KQI only needs to traverse every node in the graph once in the preparation stage, and then KQI can be calculated with a constant complexity. Therefore, the algorithm complexity of KQI is more stable (S4 Text in S1 File).
4. **Additivity.** KQI is based on entropy, the difference between Shannon entropy and structural entropy, so KQI inherits the additivity of entropy while PageRank does not. For nodes in the network, KQI can be aggregated by summing up any combination meaningfully (Fig 3F–3H).

In network analysis, several measures of entropy are available to study information content [42–44]. Although KQI shares the same basis as them in information theory, the essence of KQI is the quantification of structuration. Suppose a network contains two parts: unstructured information I and structure K. Network entropies that focus on specific properties of a graph (adjacency matrix, degree distribution, etc.) tend to omit quantification of structuration, i.e., they quantify I+o(K). Entropies of network ensembles adequately quantify network complexity, i.e., quantify I+K. Thus, entropies of network ensembles are also available for knowledge quantification, as long as K is separated out, which is still an open question.

There are limitations to our approach. For example, the latest publication is at a disadvantage over the competition. Although we could mitigate this by simply setting the weight of the citation to decay over time, it would still be unfair for articles that have not yet received citations. In addition, the value of citation is not considered, which is related to whether it truly represents the inheritance and development of knowledge from article to article. In the future, better results can be obtained by building more accurate datasets.

This study introduces a new method of KQI for the quantification of knowledge among citation networks. Leveraging the same metric, KQI is manifested to have power in digging out influential articles, researchers, institutions, etc. that might not be precisely portraited by those aforementioned measures. Our methodology can be applied generally to quantify the knowledge of directed acyclic knowledge flow graph, and to set the preliminary stage for the quantification of knowledge. In addition, an interesting application is to estimate the value of a network dataset through KQI without any further prediction algorithm.

## Materials and methods

### Collection of the dataset

Our academic data is all collected from Acemap [39], which is constructed using metadata retrieved and integrated from the known academic database including but not limited to Nature, Science, Elsevier, and Springer: more than 214 million pieces of literature published between 1800 and 2021, and 1.7 billion citations among them. All users can easily access the Acemap website to acquire academic articles, as well as their authors, affiliations, countries, publication years, publishers, disciplines, and references. In addition, we also collected laureates with Turing Award and Nobel Prize from official websites [45,46]. Our data collection methods used in this study comply with the terms of service of the data sources used. All source data used in the figures can be accessed in Supporting Information files.

### Construction of citation network

Using our collated database, we constructed a directed acyclic graph network, where nodes represent articles and edges represent citations. In principle, each article should have a unique release date, and only the older articles should be cited. As these out-of-sequence citations account for less than 1%, we simply remove them from the graph. For the ring appearing in the same year, though this is rare because the publication time of our collected articles is only accurate to year, we treat articles in a strongly connected component as the same.

### Construction of knowledge tree

The knowledge tree is constructed out of important knowledge inheritance relationships. In this article, the knowledge tree is constructed out of the citations of papers. Starting from each axiom (groundbreaking papers without references), we can get a knowledge development vein with it as the ancestor. When knowledge belongs to multiple parent communities, it will be split into several parts belonging to different communities, i.e., each parent makes a part of the contribution to the emergence of the new knowledge. Furthermore, we believe that everything has an origin, so we introduce an extra super root from which all the axioms come. The knowledge tree is such a knowledge inheritance structure that progresses from the super root downwards layer by layer.

### Calculation of KQI

Using the algorithm in the Supplementary Materials, we get the KQI of each node in the constructed citation network.

1. Article-level. The paper’ KQI is exactly the KQI of the node.
2. Author-level. First, the paper’s KQI is distributed equally to each author. Then, the author’s KQI is the summation of contribution to all papers of the author.
3. Affiliation-level. The affiliation’s KQI is the summation of all papers of the affiliation.
4. Country-level. The country’s KQI is the summation of all papers of the country.

All those papers for which affiliation or country information is missing, are ignored in summation at affiliation-level and country-level.

### Calculation of other metrics

Impact factor, h-index, and PageRank value are calculated based on our collated database, therefore, there may be some deviation compared to Clarivate, Google scholar, etc.

### The variant of the Gini coefficient

The Gini coefficient is usually defined based on the Lorenz curve, which depicts the proportion y of total income earned cumulatively by the bottom x proportion of the population. The Gini coefficient can be calculated by 1-2A, where A is the area under the Lorenz curve (both the x-axis and y-axis scaled from 0 to 1). Here, instead of the Lorenz curve, the distribution of the resampled part of the data is taken for the variant of the Gini coefficient. Since the resampled distribution can break the 45-degree line, the variant of the Gini coefficient takes the range from -1 to 1 instead of from 0 to 1 as originally. 0 still represents absolute equality, while -1 and 1 represent absolute inequality in different directions.

## Supporting information

S1 Data (RAR)

S1 File (PDF)
