# Supplementary material for: Quantifying knowledge from the perspective of information structurization
Source: PLoS One. 2023 Jan 4;18(1):e0279314. doi: 10.1371/journal.pone.0279314 (PMC9812334; doi:10.1371/journal.pone.0279314)
Supplement: S1 File — (PDF) [file pone.0279314.s002.pdf]

Quantifying Knowledge from the Perspective of Information Structurization

Xinbing Wang[1]*, Huquan Kang[1], Luoyi Fu[1], Ling Yao[2], Jiaxin Ding[1], Jianghao Wang[2], Xiaoying Gan[1], Chenghu Zhou[2], John E. Hopcroft[3]

1 Shanghai Jiao Tong University, Shanghai 200240, China.
2 State Key Laboratory of Resources and Environmental Information System, Institute of Geographic Sciences and Natural Resources Research, Chinese Academy of Sciences, Beijing 100101, China.
3 Cornell University, Ithaca, NY, 14853, USA.

These authors contributed equally to this work.
* xwang8@sjtu.edu.cn

Supporting information

S1 Text. Structural Entropy. Structure entropy [1] is proposed by Angsheng Li in 2016 to measure the structural information and complexity of the network. Different from Shannon entropy, which only considers probability distribution, structure entropy takes into account the layered community structure in the network. Its core is to use the structure as the inherent information to compress the length of the encoding.

Definition 1 (Volume [1]). *The volume of a community α is the sum of the weighted degree of all the nodes within it, defined as:*

$$V_\alpha = \sum_{i \in \alpha} d_i,$$

where d_i is the weighted degree of node i.

Definition 2 (One-Dimensional Structural Information [1]). *The one-dimensional structural information of connected and undirected graphs is defined as follows:*

$$\mathcal{H}^1(G) = H\left(\frac{d_1}{2m}, ..., \frac{d_n}{2m}\right) = -\sum_{i=1}^{n} \frac{d_i}{2m} \log_2 \frac{d_i}{2m}, \quad (1)$$

where n and m represent the number of nodes and edges of G respectively, d_i is the degree of the i-th node. This definition is essentially the Shannon entropy of the degree distribution in the graph G.

Definition 3 (Two-Dimensional Structural Information [1]). *Given a connected and undirected graph $G(V, E)$ and a partition $\mathcal{P} = \{X_1, ..., X_L\}$ of V, the two-dimensional structural information is defined as follows:*

$$\mathcal{H}^{\mathcal{P}}(G) = -\sum_{j=1}^{L} \frac{V_j}{2m} \sum_{i=1}^{n_j} \frac{d_i^{(j)}}{V_j} \log_2 \frac{d_i^{(j)}}{V_j} - \sum_{j=1}^{L} \frac{g_j}{2m} \log_2 \frac{V_j}{2m},$$

where L is the number of modules in partition $\mathcal{P}$, n_j is the number of nodes in module X_j, $d_i^{(j)}$ is the degree of the i-th node of X_j, V_j is the volume of module X_j, and g_j is

the number of edges with one endpoint in module X_j. This formula is equivalent to the code of each module plus the code of each node within the communities. That is, all nodes within a module share a common community code.

Definition 4 (High-Dimensional Structural Information [1])**.** *Suppose that $\mathcal{T}$ is a partitioning tree of a connected and undirected graph $G(V, E)$, then the high-dimensional structural information is defined as follows:*

$$\mathcal{H}^{\mathcal{T}}(G) = \sum_{\alpha \in \mathcal{T}, \alpha \neq \lambda} -\frac{g_\alpha}{2m} \log_2 \frac{V_\alpha}{V_{\alpha^-}}, \tag{2}$$

where g_α is the number of edges from nodes in T_α to nodes outside T_α, V_α is the volume of set T_α. This definition is an extension of two-dimensional structural information and reuses the code of common nodes to a greater extent.

S2 Text. Knowledge Tree. Due to the diversity and complexity of structural entropy caused by the partitioning tree, we attempt to define an interpretable tree as an example to explicitly give the expression of KQI. In order to retain the original inheritance relationship of knowledge as much as possible, we model this inheritance relationship as a Knowledge Tree, and further implement a kind of KQI by using the knowledge tree as the partitioning tree.

It is worth noting that a knowledge tree is different from a partitioning tree. Each node in the knowledge tree corresponds to both a node and a community (the structure on which the node depends) in the partitioning tree, that is, each node in the knowledge tree corresponds to a piece of knowledge. Therefore, **knowledge is used indiscriminately next to describe the nodes in the knowledge tree.** The detailed transformation of the knowledge tree and partitioning tree will be presented in Section .

We have previously claimed that academic citation networks can be considered as a directed acyclic graph, in which a knowledge inheritance tree can be obtained from either breadth-first search or depth-first search starting from any source node. Such a knowledge inheritance tree can be used as a partitioning tree, though it can not adequately represent the inheritance relationship. In this simplest case, the knowledge tree is just a tree. Although this situation hardly works in practice, it can be a good transition to understanding the general definition of the knowledge tree.

Just to make it easier for the reader, considering multiple source nodes, we first give the definition of a single inherited knowledge tree that can be applied.

Definition 5 (Single-Inherited Knowledge Tree)**.** *For a directed acyclic graph with n source nodes, at most one parent node is selected for each node, and a super root node is introduced to connect to all the n source nodes. Then we obtain the single-inherited knowledge tree.*

A single-inherited knowledge tree is reflected as essentially a forest. We say it is a knowledge tree by assuming that all the source nodes, that is, axiomatic knowledge, originate from something more primitive, which we call the super root node. A single inheritance tree expresses that every knowledge comes from one and only one source. It is important to note that this assumption is different from our common sense. We expect to be able to consider multiple knowledge sources and multiple different partitioning trees simultaneously. In order to make full use of the knowledge structure, we introduce the multiple-inherited knowledge tree.

Definition 6 (Multiple-Inherited Knowledge Tree)**.** *For a directed acyclic graph with n source nodes, multiple important parent nodes are selected for each node, and a super*

root node is introduced to connect to all the n source nodes. Then we obtain the multiple-inherited knowledge tree, or for short, knowledge tree.

Because we just give the definition straight away above, a multiple-inherited knowledge tree does not look like an actual tree. We will go into more detail about what kind of tree does it represents.

Decomposing a knowledge tree into an ordinary tree is done by diving nodes from leaves to roots. One observation is that loops in the knowledge tree are impossible, which ensures that decomposition is completed in finite steps. Regardless of the super root node, the knowledge tree should be a forest composed of n trees, but there is some overlap between the n trees that we do not want. The overlap between the trees comes from the crossover, that is, the in-degree of a node is greater than one. Crossover on node v indicates that the knowledge sub-tree starting from v belongs to the common result of multiple parent knowledge (nodes), each of which occupies a certain proportion. Thus, decomposing is to divide each node and its sub-tree into parts equal to in-degree, and we will get n ordinary trees. In the same way as a single-inherited knowledge tree, when we introduce the root node, the knowledge tree will look like an actual tree.

Here we can find that there seem to create more nodes in the knowledge tree. To explain this, we need to introduce a concept in the multiple-inherited knowledge tree: fragment.

Definition 7 (Fragments)**.** *Fragments are defined as new nodes created by splitting the node with multiple sources. One Fragment is a part of the node and belongs to only one source at the same time.*

Therefore, a multiple-inherited knowledge tree can consider all inheritance relationships of a graph by fragmenting the knowledge. Obviously, there is a trade-off between accuracy and efficiency, when we taking into account several important pieces of knowledge. In particular, an untreated directed acyclic graph can itself be considered as a knowledge tree, which completely retains all structural information in the graph with all the parent knowledge (nodes) matters. We can adjust the knowledge tree selection according to the actual requirements of different tasks.

S3 Text. KQI Formulation. Next, we will use the proposed knowledge tree as a partitioning tree to explicitly derive the formula of KQI. As with the introduction of the knowledge tree, we will discuss in detail the formula of KQI and its rationality by using the simplest single inherited knowledge tree and then extend the conclusion to the general knowledge tree.

KQI in Single-Inherited Knowledge Tree

Because a single-inherited knowledge tree is simply an ordinary tree and the knowledge in it will not be divided into multiple fragments, our discussion of formulas will be greatly simplified.

We know that the partitioning tree is a hierarchy of nodes, a tree of virtual existence. Each non-leaf node in the partitioning tree represents a community of nodes, and only the leaf nodes actually exist. Although each node in the knowledge tree corresponds to one of real existence, we say that the knowledge tree can be used as a partitioning tree because the knowledge tree naturally represents the structure of knowledge. Ancestral knowledge each forms its own knowledge community, much like the hierarchical community of the partitioning tree. The partitioning tree represented by the knowledge tree looks like this:

- All nodes in the knowledge tree are leaf nodes in the partitioning tree.

- All non-leaf nodes in the knowledge tree are duplicated as non-leaf nodes in the partitioning tree.
- Each node v has $d_v^{out}+1$ sub-communities, that is d_v^{out} derived knowledge communities plus knowledge v itself.

Using the knowledge tree as a partitioning tree in this way, we give the formula for KQI.

Theorem 1 (KQI in Single-Inherited Knowledge Tree). *Given single-inherited knowledge tree $\mathcal{T}$, the KQI expression for the node representing community α is as follows:*

$$\mathcal{K}_\alpha^{\mathcal{T}} = -\frac{V_\alpha}{W}\log_2\frac{V_\alpha}{V_{\alpha^-}}, \tag{3}$$

where V_α is the volume of community α, W is the graph size and α^- is the parent community of α.

Proof of Theorem 1

Lemma 1.1. *Any partitioning tree $\mathcal{T}$ of height h can be reconstituted as $\mathcal{T}'$ to satisfy the following conditions: 1) For any height $1 \le h_i \le h$, the communities $\alpha_1, ..., \alpha_{n_i}$ represented by all n_i nodes of height h_i are mutually exclusive, and $\bigcup_{1\le j\le n_i}\alpha_j = S$, where S is the set of all leaf nodes. 2) Before and after the reconstitution, the entropy of the original nodes and the overall entropy of the partitioning tree remains unchanged. We say that $\mathcal{T}$ and $\mathcal{T}'$ are equivalent.*

Proof of lemma 1.1. The proof of this lemma is very simple. Since the partition tree is a tree, it must satisfy the mutual exclusion. And the layer of height 1 must satisfy $\bigcup_{1\le j\le n_1}\alpha_j = S$. Then, if the layer of height 2 has $\bigcup_{1\le j\le n_2}\alpha_k = S'$ and $S' \ne S$, create a copy α' in the second layer for each $\alpha \in S - S'$ in the first layer. In this process, the entropy of α is transferred to α' and the entropy of α becomes 0, where the whole thing stays the same. Then go on to the layer of height 3 and so on until all the layers are satisfied. □

Proof of theorem 1. Suppose the knowledge tree $\mathcal{T}$ of height h is selected as the partitioning tree. For one node $\alpha \in \mathcal{T}$, i.e. a community of V, denote V_α as volume of community α, and g_α as boundary of community α. More rigorously,

$$V_\alpha = \sum_{v\in\alpha} d_v^{out}, \quad g_\alpha = \sum_{(i,j)\in E, i\notin\alpha, j\in\alpha} w_{ij}.$$

Denote the parent of α in $\mathcal{T}$ as α^-, the parent of α^- as $\alpha^{(-2)}$, and so on. The structure entropy in KEG can be written as:

$$\mathcal{H}^{\mathcal{T}} = \sum_{\alpha\in\mathcal{T}} -\frac{g_\alpha}{W}\log_2\frac{V_\alpha}{V_{\alpha^-}},$$

where $W = \sum_{(i,j)\in E} w_{ij}$.

According to Lemma 1.1, we can divide the nodes in $\mathcal{T}$ into h layers, denoted as $l_i (1 \le i \le h)$, and we have $\forall 1 \le i \le h, \bigcup_{\alpha\in l_i}\alpha = V$. Thus, we can rewrite the expression for structure entropy as follows:

$$\mathcal{H}^{\mathcal{T}} = \sum_{1\le i\le h}\sum_{\alpha\in l_i} -\frac{g_\alpha}{W}\log_2\frac{V_\alpha}{V_{\alpha^-}}.$$

As boundary of community α, g_α can also be rewritten as: $g_\alpha = \sum_{v\in\alpha} d_v^{in} - V_\alpha$. Then the formula becomes two parts:

$$\begin{aligned} FirstTerm &= \sum_{1\le i\le h}\sum_{\alpha\in l_i} -\frac{\sum_{v\in\alpha} d_v^{in}}{W}\log_2\frac{V_\alpha}{V_{\alpha^-}} \\ &= \sum_{1\le i\le h}\sum_{\alpha\in l_i}\sum_{v\in\alpha} -\frac{d_v^{in}}{W}\log_2\frac{V_{v(-i+1)}}{V_{v(-i)}} \\ &= \sum_{v\in V} -\frac{d_v^{in}}{W}\sum_{1\le i\le h}\log_2\frac{V_{v(-i+1)}}{V_{v(-i)}} \\ &= \sum_{v\in V} -\frac{d_v^{in}}{W}\log_2\frac{V_v}{V_{v(-h)}} \end{aligned}$$

where $V_v = d_v^{out}, V_{v(-h)} = W$. The first term is exactly Shannon entropy of graph. So, we have

$$\mathcal{K}^{\mathcal{T}} = SecondTerm = \sum_{1\le i\le h}\sum_{\alpha\in l_i} -\frac{V_\alpha}{W}\log_2\frac{V_\alpha}{V_{\alpha^-}}.$$

Applying above lemma again, we give the formula of KQI as follows:

$$\mathcal{K}^{\mathcal{T}} = \sum_{\alpha\in\mathcal{T}} -\frac{V_\alpha}{W}\log_2\frac{V_\alpha}{V_{\alpha^-}}.$$

The proof is completed. □

Interpretation of KQI formula

Next, let us understand what KQI means from the (3).

At the micro-level, in terms of encodings, each knowledge in the knowledge tree, like a structural pivot, plays a role in encoding reuse. KQI is a measure of how many encodings of each knowledge can be reused. The degree of reuse is expressed as the encoding length ($-\log_2 \frac{V_\alpha}{V_{\alpha^-}}$) times the probability of reuse ($\frac{V_\alpha}{W}$). It is worth noting that both the encoding length and the probability of reuse are greater than or equal to 0. This is because all variables are non-negative and $V_\alpha < V_{\alpha^-}$ is always maintained, as guaranteed by the definition of volume. Therefore, the KQI is also greater than or equal to 0. This property also holds in the generalized KQI in the later section.

At the macro-level, the structure is a form of organization, such as a hierarchy and a network. A knowledge tree, equivalent to multilevel classification, is a hierarchical structure based on knowledge evolution. The difference between Shannon entropy and structure entropy strips away discrete information other than the structure itself. When we use the knowledge tree as the partitioning tree, KQI only retains the structure of the knowledge tree. In this sense, KQI can be used as an expression of how strongly structured the knowledge is.

Starting with the concept of knowledge, KQI is related to acceptability ($\frac{V_\alpha}{W}$) and dependability ($-\log_2 \frac{V_\alpha}{V_{\alpha^-}}$). Acceptability refers to whether knowledge is recognized, i.e. how much knowledge is inherited directly or indirectly from that knowledge. Dependability refers to whether the source of knowledge is equally or more recognized, i.e. how fully the parents can support the generation of the knowledge. Acceptability and dependability, elements of scientific knowledge [2,3], correspond to the first and second terms of the (3), as well as to truth and justification as we talked about earlier in JTB theory [4].

We can find the higher the probability of the random walk reaching the node, or the higher the level in the partitioning tree, the more repetitive encoding is reduced and the more strongly structured the node is. In other words, the more connectivity and the more subsequent knowledge, the higher KQI. Intuitively, under the definition of KQI, knowledge with higher KQI is developed from very solid knowledge and can form its own system. If knowledge is derived from the knowledge that is not recognized, its KQI is small and can be understood as unacknowledged.

Generalized KQI in Knowledge Tree

Now we have the formula for KQI in the simplest single-inherited knowledge tree. In this part, we will extend KQI to a general knowledge tree.

In the discussion of knowledge tree, we know that a knowledge tree can also be regarded as a real tree. But the process of decomposing into a tree can lead to some splitting of knowledge. So if we want to get the KQI of one knowledge, we have to consider all the fragments that are formed by splitting that knowledge. Here we give the definition of KQI in the general knowledge tree.

Definition 8 (KQI in Knowledge Tree)**.** *Given knowledge tree $\mathcal{T}$ and denoted S_α as the set of all fragments of α, the KQI of α is defined as:*

$$\mathcal{K}_\alpha^\mathcal{T} = \sum_{\alpha_i \in S_\alpha} -\frac{V_{\alpha_i}}{W} \log_2 \frac{V_{\alpha_i}}{V_{\alpha_i^-}}. \tag{4}$$

If we use (3) directly, this is a fairly complex process because the number of fragments is exponentially related to the depth of knowledge. If each knowledge originated from two parents, the k-th generation of knowledge will be split into 2^k fragments. Here we present a method equivalent to the above calculation, but with much less complexity.

Theorem 2 (More Efficient Equivalent KQI in Knowledge Tree)**.** *Given knowledge tree $\mathcal{T}$, the KQI expression defined in Definition 8 is equivalent to following expression:*

$$\mathcal{K}_\alpha^\mathcal{T} = -\sum_{1 \le i \le d_\alpha^{in}} \frac{V_\alpha^\mathcal{T}}{d_\alpha^{in} W} \log_2 \frac{V_\alpha^\mathcal{T}}{d_\alpha^{in} V_{\alpha_i^-}^\mathcal{T}}, \tag{5}$$

in which

$$V_\alpha^\mathcal{T} = d_\alpha^{out} + \sum_{1 \le i \le d_\alpha^{out}} \frac{V_{\alpha_i^+}^\mathcal{T}}{d_{\alpha_i^+}^{in}},$$

where α_i^- represents the i-th parent of α, α_i^+ represents the i-th child of α, $V_\alpha^\mathcal{T}$ represents the sum of all the V_α of different fragments in $\mathcal{T}$. It can be proved that (5) is equivalent to (4).

Before proving that the above formulas are equivalent, let us introduce two lemmas first.

Lemma 2.1. *Assume β has n fragments $\beta_1, ..., \beta_n$, and α is a child of β, then we have $\frac{V_{\alpha_1}}{V_{\beta_1}} = ... = \frac{V_{\alpha_n}}{V_{\beta_n}}$.*

Proof of lemma 2.1. This lemma makes sense because, in our definition, all of the successive nodes of β will be split the same way when we split β. □

Lemma 2.2. *Given n-source knowledge tree $\mathcal{T}$, in which knowledge α has n fragments $\alpha_1, ..., \alpha_n$, we have $V_\alpha^\mathcal{T} = \sum_{i=1}^n V_{\alpha_i}$.*

Proof of lemma 2.2. We use inductive reasoning to prove it. (1) If knowledge α is a leaf node, α has no children and no fragments. Obviously, $V_\alpha^{\mathcal{T}} = d_\alpha^{out} = V_\alpha$. (2) Assuming that all children of α satisfy the lemma, next we explain that α also satisfies the lemma. Given α^+ that is a child of α, we say that each fragment α_i^+ is added to α_i in the same proportion $\frac{1}{d^{in}_{\alpha_i^+}}$. So sum over all the α_i^+ is the same as $\sum_{1 \le i \le d_\alpha^{out}} \frac{V^{\mathcal{T}}_{\alpha_i^+}}{d^{in}_{\alpha_i^+}}$. And the sum of the out-degree of all the fragments of α is also equal to d_α^{out}. Thus, the lemma is proved. □

Now let us prove Theorem 2.

Proof of theorem 2. Divide all the fragments of α into d_α^{in} groups, denoted as $S_{\alpha_i^-}(1 \le i \le d_\alpha^{in})$, according to α's parent. The fragment in group $S_{\alpha_i^-}$ is denoted as $\alpha_{(i,j)}(1 \le j \le |S_{\alpha_i^-}|)$. Applying lemma 1, in the same group $S_{\alpha_i^-}$, the proportion $\frac{V_{\alpha_{(i,j)}}}{V_{\alpha^-_{(i,j)}}}$ stays the same. Applying lemma 2, $\frac{V_{\alpha_{(i,j)}}}{V_{\alpha^-_{(i,j)}}} = \frac{\sum_{j=1}^{|S_{\alpha_i^-}|} V_{\alpha_{(i,j)}}}{\sum_{j=1}^{|S_{\alpha_i^-}|} V_{\alpha^-_{(i,j)}}} = \frac{V^{\mathcal{T}}_{\alpha_i}}{V^{\mathcal{T}}_{\alpha_i^-}}$. So we can sum over $\mathcal{K}^{\mathcal{T}}_{\alpha_{(i,j)}}$ in each of these groups separately.

$$
\begin{aligned}
\sum_{1 \le j \le |S_{\alpha_i^-}|} \mathcal{K}^{\mathcal{T}}_{\alpha_{(i,j)}} &= \sum_{1 \le j \le |S_{\alpha_i^-}|} -\frac{V_{\alpha_{(i,j)}}}{W} \log_2 \frac{V_{\alpha_{(i,j)}}}{V_{\alpha^-_{(i,j)}}} \\
&= \sum_{1 \le j \le |S_{\alpha_i^-}|} -\frac{V_{\alpha_{(i,j)}}}{W} \log_2 \frac{V^{\mathcal{T}}_{\alpha_i}}{V^{\mathcal{T}}_{\alpha_i^-}} \\
&= -\frac{V^{\mathcal{T}}_{\alpha_i}}{W} \log_2 \frac{V^{\mathcal{T}}_{\alpha_i}}{V^{\mathcal{T}}_{\alpha_i^-}} \\
&= -\frac{V^{\mathcal{T}}_{\alpha}}{d_\alpha^{in} W} \log_2 \frac{V^{\mathcal{T}}_{\alpha}}{d_\alpha^{in} V^{\mathcal{T}}_{\alpha_i^-}}
\end{aligned}
$$

Then when we add up all the groups, we find that the result is the same as (4). □

S4 Text. KQI Algorithm. In this part, we give the algorithm of KQI, whose input is a directed acyclic graph. The algorithm consists of two stages: preparation and query. In the preparation phase, lines 1 to 3, go through all the knowledge in reversed topological order, calculate their volumes and the weight of the graph. In the query phase, lines 5 to 6, we apply (5) in the general knowledge tree to calculate the KQI.

Obviously, the complexity of the preparation phase is $O(n)$, and the complexity of the query phase is $O(1)$.

S1 Fig. Countries Ranking by KQI. According to the country of papers' first author (incomplete), the top 20 countries are listed by aggregating KQI by country, with literature marked either.

Algorithm 1: KQI

Input: Directed Acyclic Graph (V, E)

Output: KQI of each node v

for $v \in$ *TopologicalSort(V)* **do**

  $W = W + d_v^{out}$

  $vol[v] = d_v^{out} + \sum_{v \to u} \frac{vol[u]}{d_u^{in}}$

end

Function `KQI`(v)**:**

  return $\sum_{u \to v} -\frac{vol[v]/d_v^{in}}{W} \log_2 \frac{vol[v]/d_v^{in}}{vol[u]}$

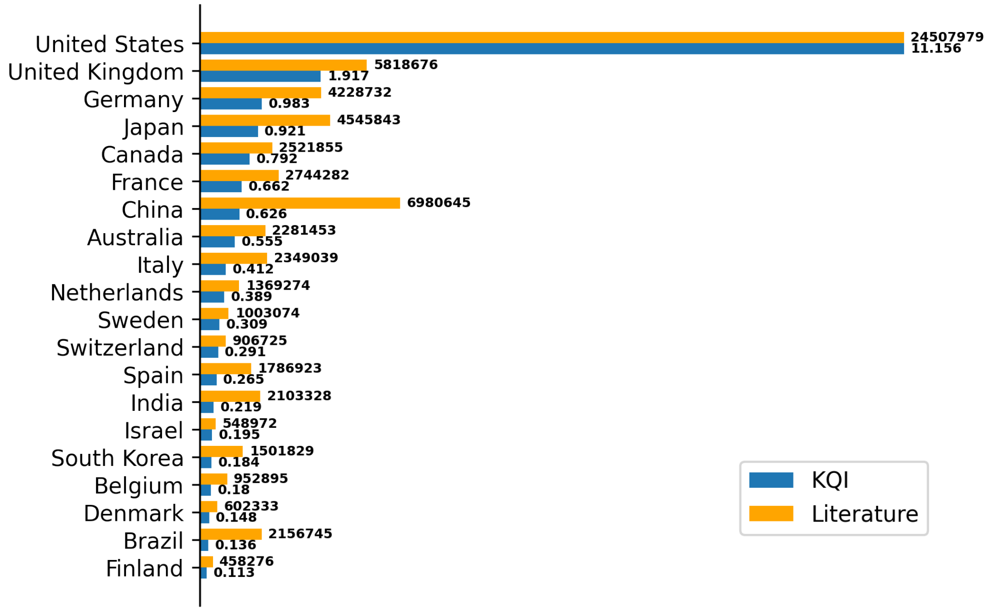

S2 Fig. Disciplines Ranking. According to the research topics of papers (a paper can correspond to more than one topic), the top 50 disciplines are listed by aggregating KQI by discipline.

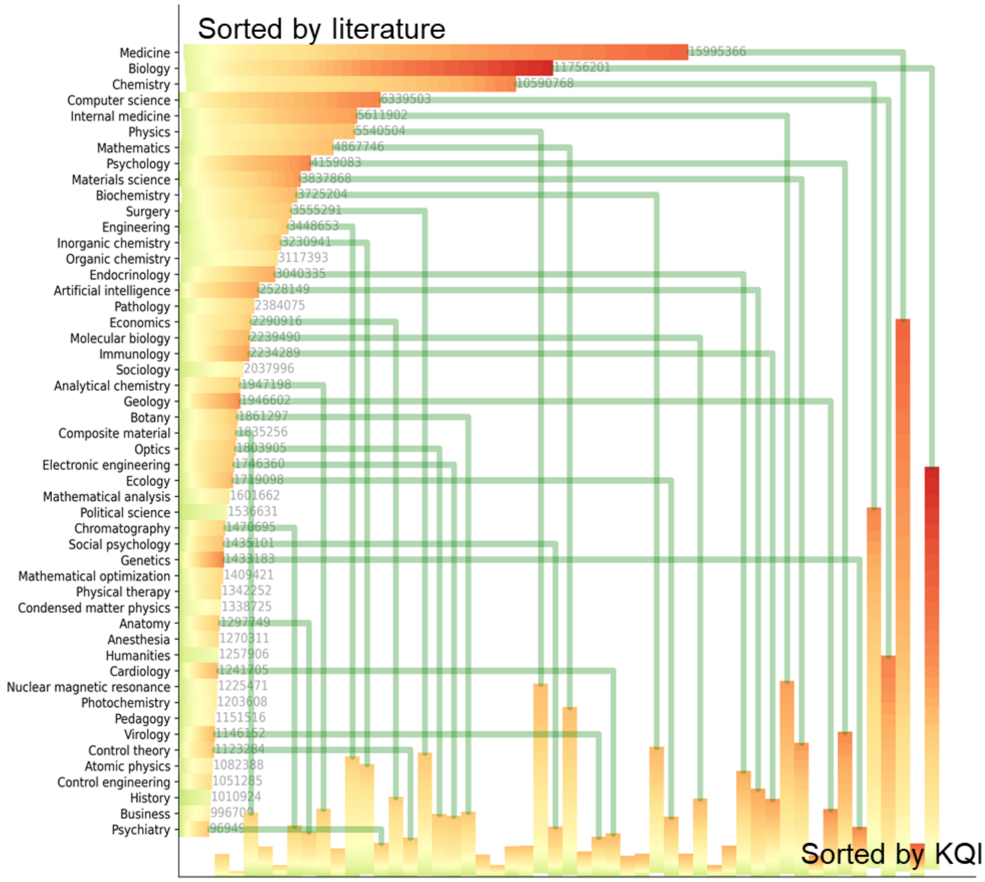

S3 Fig. KQI of Turing Award winners. In this experimental dataset in computer science, there are 6,253,122 authors. By 2020, there are 74 Turing Award winners, and thirty percent of the top 50 authors according to KQI are Turing Award winners, while the remaining 70 authors are also highly influential and receive honors such as the IEEE John von Neumann Medal, MacArthur Fellows Program, or MacArthur Fellowship, Frederick W. Lanchester Prize, etc. We single out the top 10,000 authors (0.16%) according to KQI (only first author concerned) and find 71 Turing Award winners (96%) among them. The remaining three authors are Alan Perlis, James H. Wilkinson, and Kristen Nygaard. In the case of Alan Perlis, due to some objective factors, we do not have his representative works in this dataset. For James H. Wilkinson, his outstanding contribution in numerical analysis is classified into the field of mathematics by our dataset. Kristen Nygaard, who co-invented object-oriented programming and the Simula programming language with Ole-Johan Dahl, is listed as the second author. Kristen Nygaard falls behind in the rankings because we only consider the first author when dealing with the rankings.

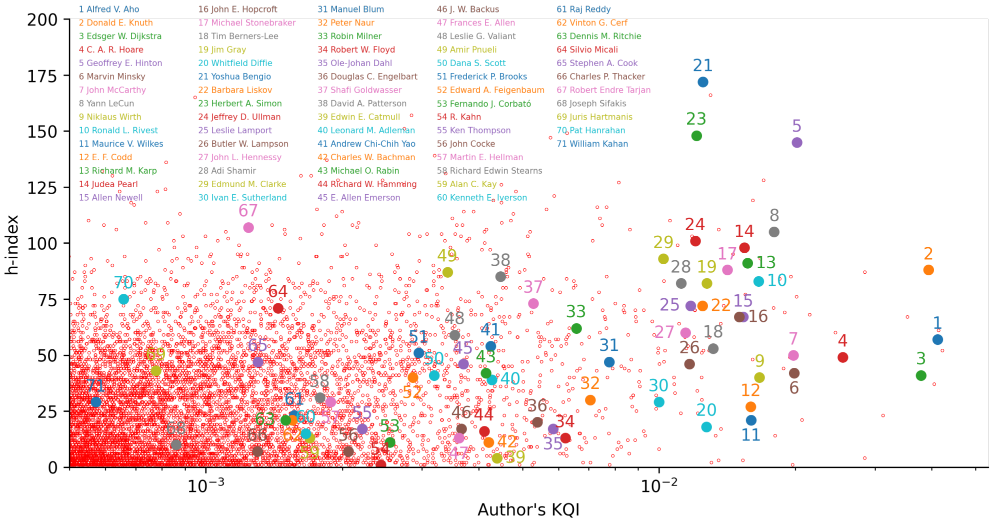

S4 Fig. KQI of Nobel prize in Economic Sciences winners. This experiment is conducted in economics, find that KQI singles out 85 (98%) of the 86 winners of the Nobel Memorial Prize in Economic Sciences, by zooming in on the part of high KQI (top 10,000 authors), which is far beyond the competence of h-index. The only author not on the list, Leonid Vitaliyevich Kantorovich, is known for linear programming, which is classified by this dataset in the mathematical field.

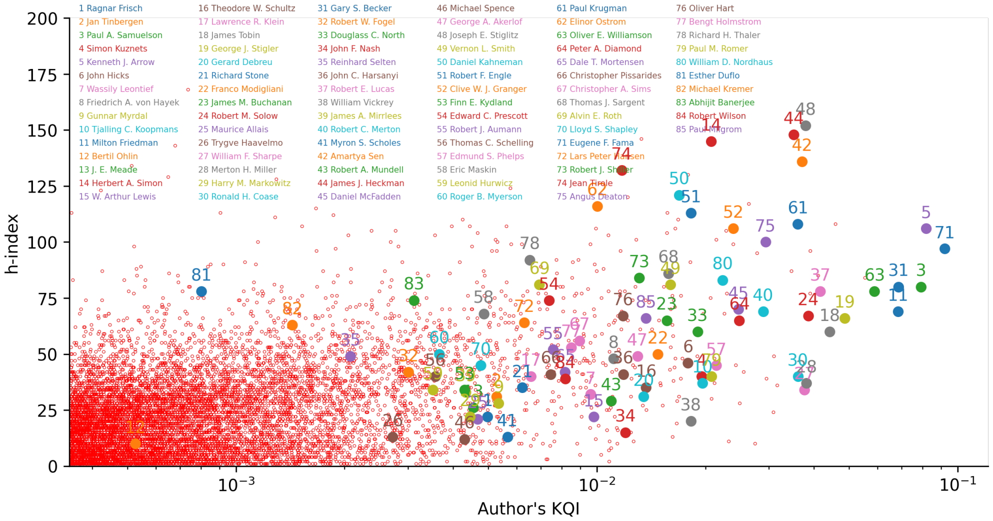

S5 Fig. Tremendous scientific productivity. a, Growth in the number of literatures. The number of literatures showed an accelerated growth with the increase of years. The total number of literatures in 2020 (198 million) has reached 3.3 times that of 2000 (61 million) and 15 times that of 1970 (13 million). b, Quantity distribution of literatures in different disciplines. Of all 292 second-level disciplines, more than 39% have more than 1 million literatures by 2020 and almost all of them have more than 100 thousand literatures.

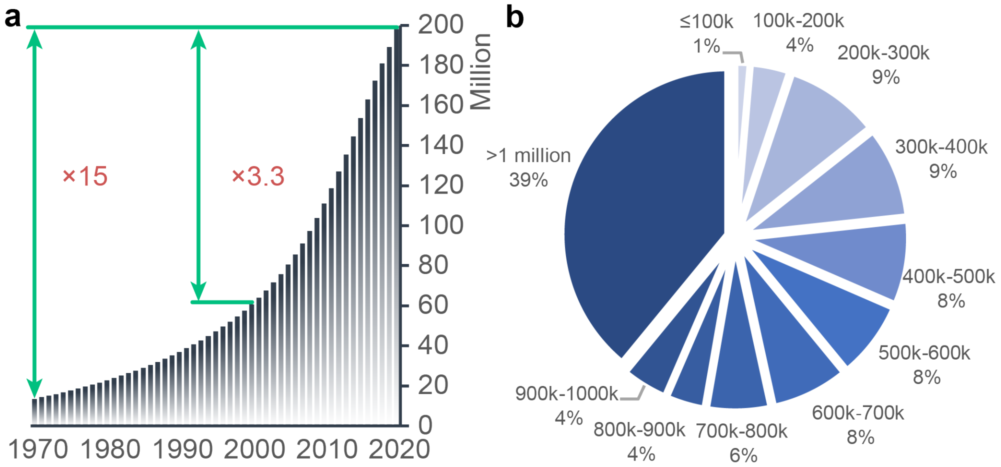

S1 Table. Papers Ranking by KQI. Building a citation network for all papers prior to 2021, the top 100 papers are listed using KQI.

S2 Table. Authors Ranking by KQI. Assuming that all authors of a paper contribute equally, the KQI of a paper is equally distributed among the authors. The top 100 authors are listed by aggregating KQI using addition. Authors who have won the Nobel Prize or the Turing Award are marked with an asterisk.

S3 Table. Affiliations Ranking by KQI. According to the affiliation of papers' first author (incomplete), the top 100 affiliations are listed by aggregating KQI by affiliations.

References

1. Li A, Pan Y. Structural information and dynamical complexity of networks. IEEE Transactions on Information Theory. 2016;62(6):3290–3339.
2. Wu L, Wang D, Evans JA. Large teams develop and small teams disrupt science and technology. Nature. 2019;566(7744):378–382.
3. Uzzi B, Mukherjee S, Stringer M, Jones B. Atypical combinations and scientific impact. Science. 2013;342(6157):468–472.
4. Steup M. The analysis of knowledge. Stanford encyclopedia of philosophy. 2007;.

S1 Table. Papers Ranking by KQI.

No.	Title	Year	KQI ($\times 10^{-4}$)
1	Molecular cloning : a laboratory manual	1989	74.920
2	Atlas of protein sequence and structure	1965	39.744
3	A mathematical theory of communication	1948	33.470
4	An introduction to probability theory and its applications	1950	32.119
5	Statistical Power Analysis for the Behavioral Sciences	1969	30.003
6	Statistical Methods for Research Workers	1925	29.836
7	Nonparametric statistics for the behavioral sciences	1956	27.698
8	THE COLORIMETRIC DETERMINATION OF PHOSPHORUS	1925	27.304
9	Physiological Studies of Conditional Lethal Mutants of Bacteriophage T4D	1963	27.244
10	Morphogenesis of bacteriophage T4 in extracts of mutant-infected cells	1966	26.655
11	Diagnostic and Statistical Manual of Mental Disorders	1994	26.311
12	Methods in Enzymology , Vol	1966	24.951
13	DETERMINATION OF SERUM PROTEINS BY MEANS OF THE BIURET REACTION	1949	24.760
14	Experiments in molecular genetics	1972	23.069
15	Handbook of Mathematical Functions	1966	23.037
16	The Nature of the Chemical Bond	1939	22.954
17	The Nature of Statistical Learning Theory	1995	22.355
18	Physics of semiconductor devices	1969	21.026
19	Statistical methods	1980	20.547
20	Standard methods for the examination of water and wastewater	1992	20.498
21	Gradient-based learning applied to document recognition	2001	19.709
22	Physics of Semiconductor Devices	1969	19.567
23	The Art of Computer Programming	1968	19.266
24	International tables for X-ray crystallography	1962	18.334
25	Principles of Behavior	1944	18.260
26	Principles of Optics	1959	18.142
27	Random Graphs	1985	17.572
28	CRC Handbook of Chemistry and Physics	1973	17.202
29	An Introduction to the Bootstrap	1993	17.102
30	Theory of elasticity	1934	17.078
31	Computers and Intractability: A Guide to the Theory of NP-Completeness	1979	16.522
32	R: A language and environment for statistical computing.	2014	16.444
33	Antibodies: A Laboratory Manual	1988	16.165
34	Elements of Information Theory	1991	15.561
35	Pattern Classification And Scene Analysis	1974	15.339
36	Matrix Computations	1983	15.193
37	Pattern classification and scene analysis	1973	15.178
38	Parallel Distributed Processing: Explorations in the Microstructure of Cognition: Volume 1: Foundations	1986	15.171
39	Introduction to solid state physics	1953	15.129
40	Genetic algorithms in search, optimization, and machine learning	1989	14.743
41	The Genetical Theory of Natural Selection	1930	14.468
42	Ca2+-stimulated ribonuclease. A new marker enzyme of differentiated rat mammary tissues.	1979	14.443
43	Applied Logistic Regression	1989	14.398
44	Quantum Computation and Quantum Information	2000	14.387
45	Stochastic processes	1953	14.329
46	A RATING SCALE FOR DEPRESSION	1960	14.124
47	Classification and Regression Trees.	1984	13.887
48	The advanced theory of statistics	1958	13.881
49	A new method for sequencing DNA	1977	13.847
50	Evaluation of survival data and two new rank order statistics arising in its consideration.	1966	13.807

Continued on next page

No.	Title	Year	KQI ($\times 10^{-4}$)
51	ON TYROSINE AND TRYPTOPHANE DETERMINATIONS IN PROTEINS	1927	13.806
52	Applied Regression Analysis	1967	13.623
53	Computational crystallography edited by D. Sayre	1983	13.484
54	Quantum Mechanics	1961	13.461
55	Time Series Analysis: Forecasting and Control	1976	13.179
56	Constitution of Binary Alloys	1958	13.034
57	Phylip - phylogeny inference package (version 3	1989	12.751
58	Statistical Methods for Rates and Proportions	1975	12.606
59	Mathematical analysis of random noise	1944	12.588
60	Learning internal representations by error propagation	1986	12.572
61	Qualitative analysis of proteins: a partition chromatographic method using paper	1944	12.510
62	A METHOD FOR THE SOLUTION OF CERTAIN NON – LINEAR PROBLEMS IN LEAST SQUARES	1944	12.486
63	Theory of Games and Economic Behavior	1944	12.475
64	MOLSCRIPT: a program to produce both detailed and schematic plots of protein structures	1991	12.437
65	Table of Integrals, Series, and Products	1943	12.375
66	The Design of Experiments	1935	12.370
67	Statistical methods for rates and proportions	1981	12.283
68	Summary of the second report of the National Cholesterol Education Program (NCEP) Expert Panel on Detectio...	1993	12.132
69	The Design and Analysis of Computer Algorithms	1974	12.019
70	THE BIURET REACTION I. THE BIURET REACTION OF ACID IMIDES OF THE BARBITURIC ACID TYPE	1928	11.956
71	The Determination of Enzyme Dissociation Constants	1934	11.790
72	The pharmacological basis of therapeutics	1941	11.779
73	Principles of Polymer Chemistry	1953	11.775
74	The Advanced Theory of Statistics	1963	11.715
75	A Theory of Cognitive Dissonance	1957	11.545
76	ON PHOSPHOTUNGSTIC-PHOSPHOMOLYBDIC COMPOUNDS AS COLOR REAGENTS	1912	11.484
77	Modifications in the colorimetric determination of the plasma proteins by the Folin phenol reagent.	1936	11.444
78	The Mathematical Theory of Communication	1950	11.394
79	An Introduction to Multivariate Statistical Analysis	1958	11.383
80	Molecular theory of gases and liquids	1954	11.370
81	Methods of enzymatic analysis	1963	11.350
82	The Statistical Analysis of Failure Time Data	1980	11.336
83	Numerical Recipes in C	1988	11.323
84	Introduction to Algorithms	1990	11.293
85	The Mathematics of Diffusion	1956	11.250
86	Estimating the Dimension of a Model	1978	11.230
87	The adaptation of the Beckman spectrophotometer to measurements on minute quantities of biological materials.	1946	11.222
88	Methods in yeast genetics	1979	11.206
89	Handbook of biochemistry : Selected data for molecular biology	1968	11.123
90	Digital Communications	1983	11.105
91	Electrodynamics of Continuous Media	1960	11.090
92	The mathematical theory of plasticity	1950	11.058
93	Absorption and Scattering of Light by Small Particles	1983	11.053
94	Phenolic Hydroxyl Ionization in Proteins. I. Bovine Serum Albumin1-3	1952	11.049
95	Disk electrophoresis of basic proteins and peptides on polyacrylamide gels.	1962	11.033
96	The Statistical Analysis of Failure Time Data	1982	11.018
97	Intermolecular and Surface Forces	1985	11.013
98	Principles and procedures of statistics.	1960	10.962
99	Interference of sodium ethylenediaminetetraacetate in the determination of proteins and its elimination	1966	10.946
100	Regression models and life tables (with discussion	1972	10.892

S2 Table. Authors Ranking by KQI.

No.	Name	KQI ($\times 10^{-4}$)	No.	Name	KQI ($\times 10^{-4}$)
1	R. A. Fisher	85.399	51	Paul J. Flory*	22.396
2	Claude E. Shannon	65.644	52	John W. Tukey	22.094
3	Linus Pauling*	49.194	53	M. R. Garey	21.977
4	William Feller	48.469	54	Robert C. Weast	21.771
5	M. G. Kendall	41.972	55	Clark L. Hull	21.562
6	D. R. Cox	40.170	56	Amartya Sen*	21.409
7	Tom Maniatis	39.914	57	Harold Hotelling	21.074
8	Albert Bandura	39.308	58	Teuvo Kohonen	20.925
9	Jacob Cohen	37.930	59	William H. Press	20.840
10	B. F. Skinner	37.211	60	Pierre Bourdieu	20.656
11	Kenneth J. Arrow*	34.564	61	M. S. Shur	20.583
12	Oliver E. Williamson*	34.299	62	S. M. Sze	20.441
13	Karl Pearson	33.571	63	David R. Lide	20.296
14	Herbert A. Simon**	33.486	64	Douglas G. Altman	20.034
15	Joseph Sambrook	31.892	65	Michael C. Jensen	19.944
16	L. L. Thurstone	31.157	66	Charles Darwin	19.887
17	George W. Snedecor	30.914	67	S. Chandrasekhar	19.771
18	Edward F. Fritsch	30.678	68	Joseph E. Stiglitz*	19.584
19	Vladimir Vapnik	30.060	69	Daniel Kahneman*	19.327
20	Steven Weinberg*	29.975	70	J. P. Guilford	19.192
21	Lotfi A. Zadeh	29.616	71	Nathan Mantel	19.133
22	Otto Folin	29.605	72	Allen Newell*	18.992
23	Jean Piaget	29.018	73	O. Smithies	18.962
24	Gary S. Becker*	28.974	74	Charles Tanford	18.933
25	Milton Friedman*	28.893	75	Patrick Billingsley	18.891
26	David E. Goldberg	28.689	76	H. J. Eysenck	18.879
27	T.W. Anderson	28.645	77	Anthony Giddens	18.727
28	Sidney Siegel	28.353	78	Y. Subbarow	18.673
29	Sigmund Freud	28.161	79	Norbert Wiener	18.661
30	Bradley Efron	27.989	80	John H. Holland	18.606
31	Janet B. W. Williams	27.942	81	Jeffrey D. Ullman*	18.559
32	Irving Langmuir*	27.936	82	Stephen P. Timoshenko	18.438
33	George E. P. Box	27.329	83	Geoffrey E. Hinton*	18.435
34	Paul A. Samuelson*	27.146	84	Clive W. J. Granger*	18.356
35	Geoffrey Ingram Taylor	26.788	85	Richard Phillips Feynman	18.308
36	Sewall Wright	26.669	86	George J. Stigler*	18.251
37	Donald E. Knuth*	26.582	87	Cyrus H. Fiske	18.245
38	John von Neumann	26.215	88	Edsger W. Dijkstra*	18.080
39	Solomon H. Snyder	26.120	89	Donald D. Van Slyke	18.078
40	Jeffrey H Miller	25.983	90	Robert M. May	17.945
41	Masatoshi Nei	25.897	91	Zvi Griliches	17.931
42	J. C. Slater	25.338	92	Albert Einstein*	17.841
43	Benoit B. Mandelbrot	25.053	93	P. A. M. Dirac*	17.780
44	Joseph Felsenstein	24.954	94	W. Shockley	17.780
45	Motoo Kimura	23.506	95	Amos Tversky	17.720
46	Noam Chomsky	23.241	96	John E. Ware	17.708
47	Robert Tibshirani	22.767	97	Dimitri P. Bertsekas	17.628
48	Richard Bellman	22.677	98	F. Sanger	17.568
49	Donald B. Rubin	22.674	99	Calyampudi Radhakrishna Rao	17.495
50	Saul A. Teukolsky	22.599	100	Leon Festinger	17.411

S3 Table. Affiliations Ranking by KQI.

No.	Name	KQI ($\times 10^{-4}$)	No.	Name	KQI ($\times 10^{-4}$)
1	Harvard University	4314.055	51	IBM	644.219
2	Stanford University	3073.220	52	Carnegie Mellon University	623.605
3	National Institutes of Health	2375.006	53	Purdue University	606.947
4	Massachusetts Institute of Technology	2207.661	54	McGill University	603.696
5	University of California, Berkeley	2193.094	55	Boston University	594.303
6	University of Michigan	1845.644	56	University of California, Irvine	591.930
7	University of Cambridge	1822.393	57	University of Edinburgh	586.073
8	Columbia University	1798.909	58	University of Manchester	586.070
9	University of California, Los Angeles	1782.025	59	University of Utah	580.203
10	University of Washington	1740.124	60	University of Melbourne	573.738
11	Yale University	1589.931	61	Osaka University	565.124
12	Max Planck Society	1563.456	62	Karolinska Institutet	552.533
13	University of Chicago	1499.324	63	Texas A&M University	548.657
14	University of Pennsylvania	1496.673	64	University of Rochester	540.651
15	University of Wisconsin-Madison	1409.335	65	University of Texas Southwestern Medical Center	537.137
16	Cornell University	1401.081	66	University of Iowa	537.066
17	University of California, San Diego	1373.094	67	Spanish National Research Council	526.036
18	University of Minnesota	1368.138	68	Michigan State University	523.051
19	Johns Hopkins University	1313.751	69	Case Western Reserve University	515.382
20	University of Oxford	1309.242	70	University of Virginia	512.322
21	Princeton University	1283.582	71	University of Colorado Boulder	506.716
22	New York University	1172.194	72	Vanderbilt University	506.472
23	University of California, San Francisco	1165.678	73	University of Copenhagen	500.675
24	University of Toronto	1161.220	74	Lund University	499.341
25	Duke University	1138.047	75	Brigham and Women's Hospital	493.569
26	Centre national de la recherche scientifique	1115.222	76	Rockefeller University	473.213
27	University of Illinois at Urbana–Champaign	1078.021	77	University of Sydney	472.606
28	Northwestern University	1052.433	78	Brown University	467.860
29	University of Texas at Austin	1034.175	79	Arizona State University	463.401
30	Washington University in St. Louis	981.890	80	University of Amsterdam	460.407
31	University College London	971.396	81	King's College London	456.631
32	University of Southern California	957.277	82	University of California, Santa Barbara	455.662
33	University of Tokyo	937.604	83	Hebrew University of Jerusalem	455.032
34	Chinese Academy of Sciences	903.581	84	Ludwig Maximilian University of Munich	448.234
35	California Institute of Technology	902.551	85	Utrecht University	447.436
36	University of North Carolina at Chapel Hill	882.683	86	University of Paris	446.511
37	University of Pittsburgh	853.370	87	Emory University	443.105
38	Pennsylvania State University	833.428	88	Katholieke Universiteit Leuven	439.741
39	Ohio State University	810.434	89	University of Bristol	435.826
40	University of California, Davis	798.666	90	University of Zurich	434.222
41	Rutgers University	759.650	91	University of Queensland	434.211
42	University of Florida	743.100	92	Australian National University	424.273
43	University of British Columbia	728.339	93	Baylor College of Medicine	420.760
44	Imperial College London	716.584	94	University of Alberta	419.484
45	French Institute of Health and Medical Research	712.008	95	University of Massachusetts Amherst	402.536
46	University of Maryland, College Park	701.468	96	Uppsala University	402.314
47	Mayo Clinic	666.846	97	Tohoku University	399.097
48	Bell Labs	664.646	98	National Institute of Standards and Technology	393.813
49	Kyoto University	662.686	99	University of Texas MD Anderson Cancer Center	393.462
50	University of Arizona	646.425	100	University of Miami	390.988
